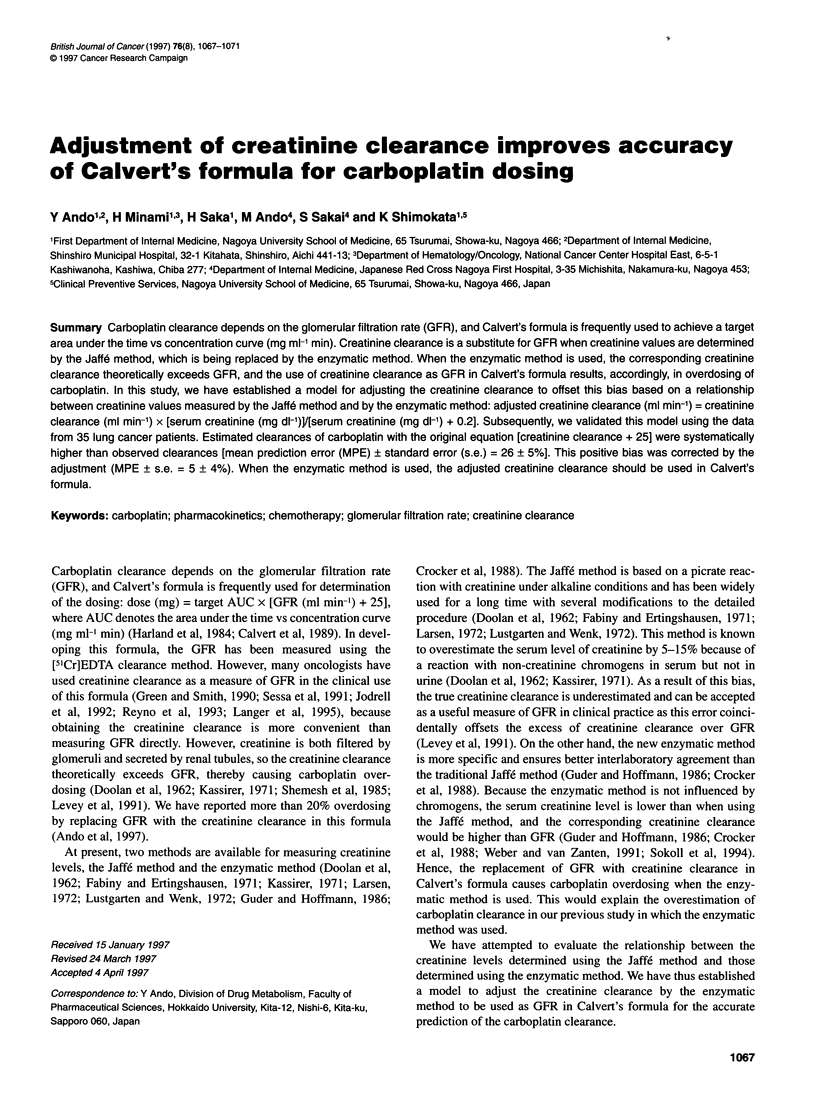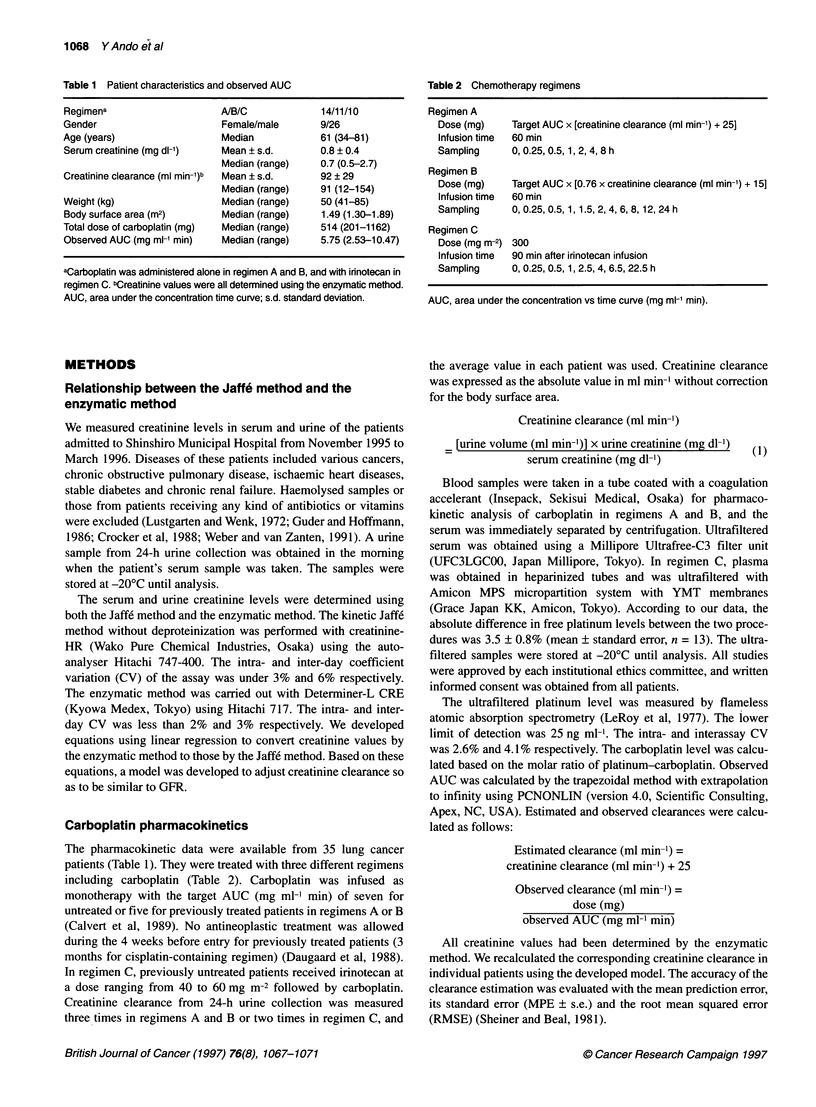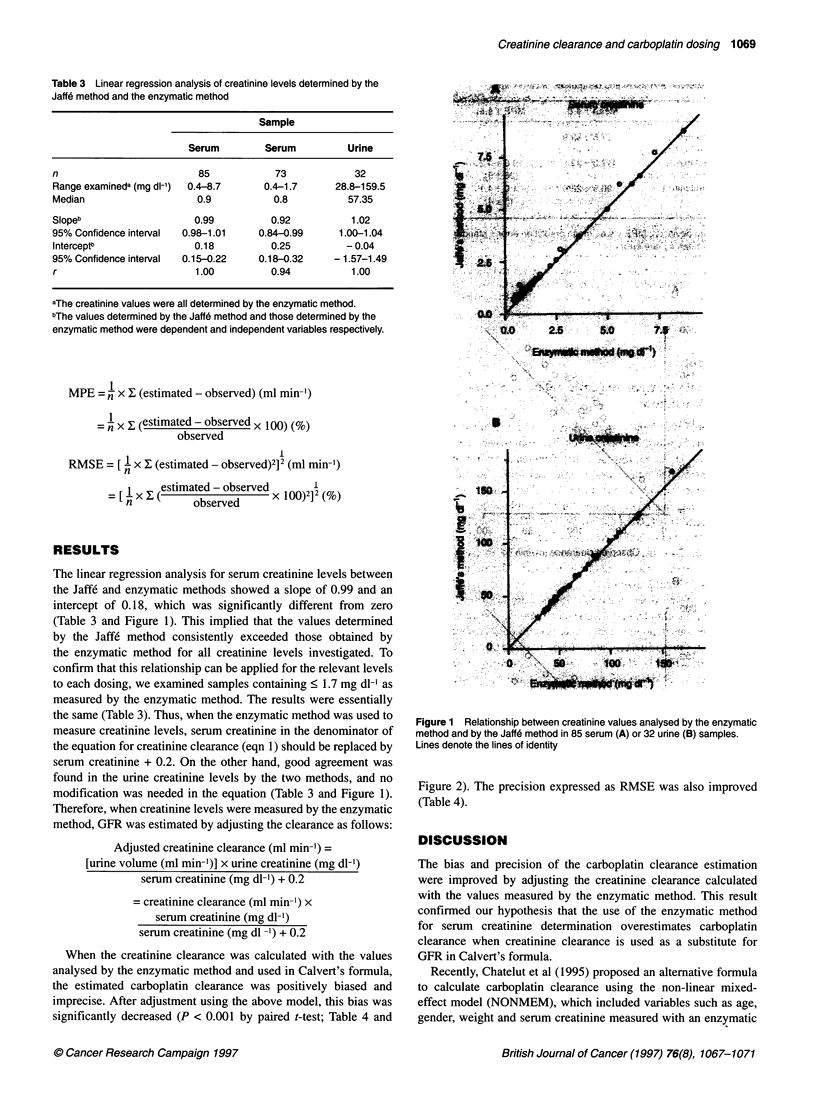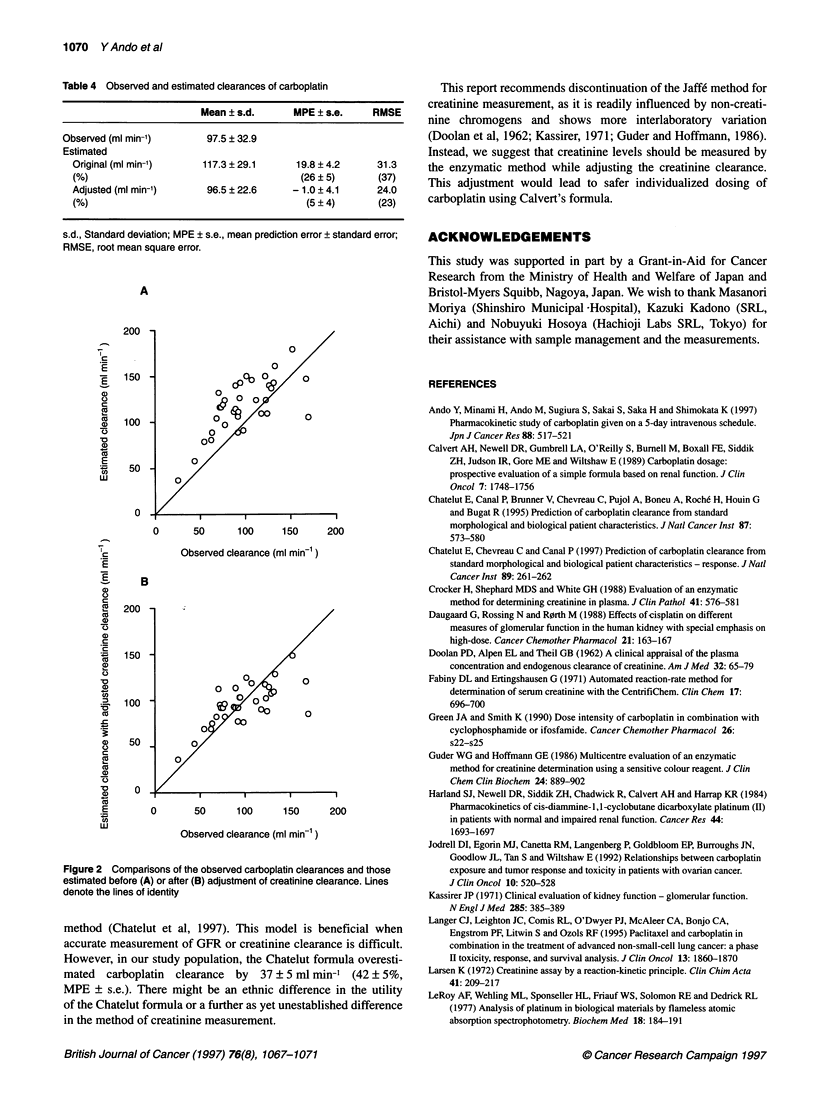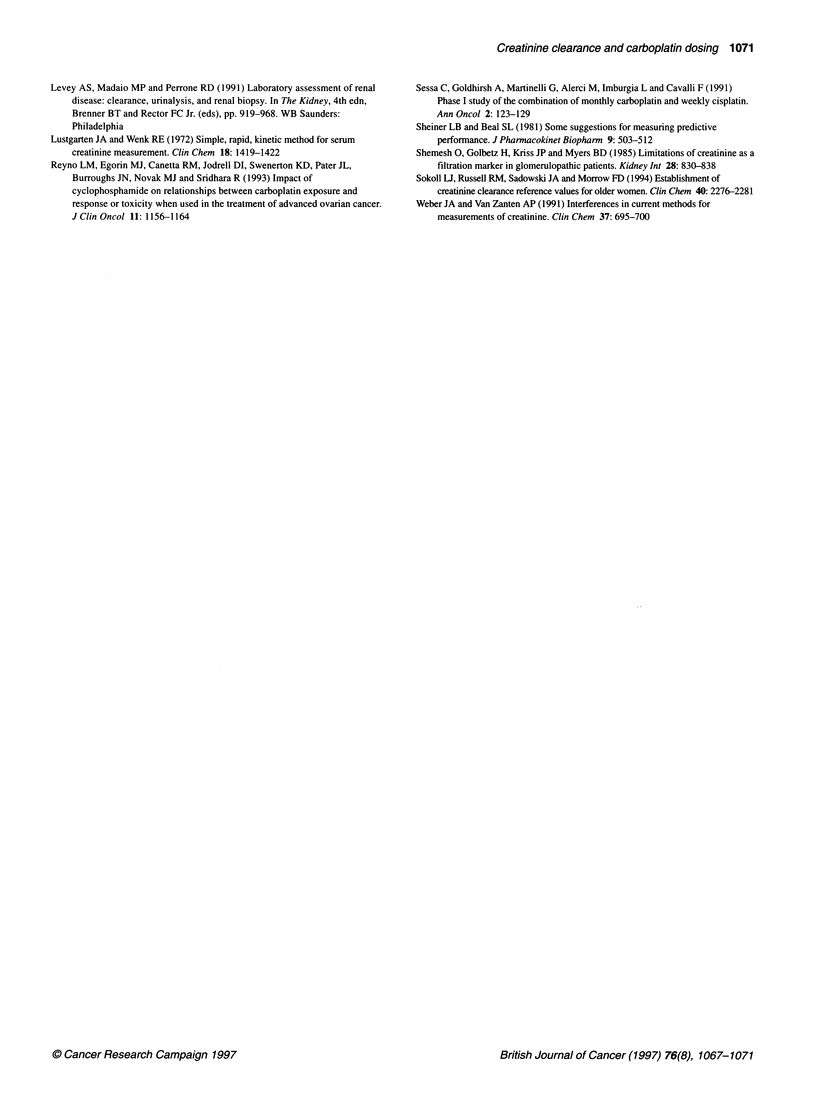# Adjustment of creatinine clearance improves accuracy of Calvert's formula for carboplatin dosing.

**DOI:** 10.1038/bjc.1997.509

**Published:** 1997

**Authors:** Y. Ando, H. Minami, H. Saka, M. Ando, S. Sakai, K. Shimokata

**Affiliations:** First Department of Internal Medicine, Nagoya University School of Medicine, Japan.

## Abstract

Carboplatin clearance depends on the glomerular filtration rate (GFR), and Calvert's formula is frequently used to achieve a target area under the time vs concentration curve (mg ml(-1) min). Creatinine clearance is a substitute for GFR when creatinine values are determined by the JaffÃ© method, which is being replaced by the enzymatic method. When the enzymatic method is used, the corresponding creatinine clearance theoretically exceeds GFR, and the use of creatinine clearance as GFR in Calvert's formula results, accordingly, in overdosing of carboplatin. In this study, we have established a model for adjusting the creatinine clearance to offset this bias based on a relationship between creatinine values measured by the JaffÃ© method and by the enzymatic method: adjusted creatinine clearance (ml min(-1)) = creatinine clearance (ml min(-1)) x [serum creatinine (mg dl(-1))]/[serum creatinine (mg dl(-1)) + 0.2]. Subsequently, we validated this model using the data from 35 lung cancer patients. Estimated clearances of carboplatin with the original equation [creatinine clearance + 25] were systematically higher than observed clearances [mean prediction error (MPE) +/- standard error (s.e.) = 26 +/- 5%]. This positive bias was corrected by the adjustment (MPE +/- s.e. = 5 +/- 4%). When the enzymatic method is used, the adjusted creatinine clearance should be used in Calvert's formula.


British Journal of Cancer (1997) 76(8), 1067–1071
© 1997 Cancer Research Campaign

### Adjustment of creatinine clearance improves accuracy of Calvert's formula for carboplatin dosing

**Y Ando[1,2], H Minami[1,3], H Saka[1], M Ando[4], S Sakai[4] and K Shimokata[1,5]**

[1]First Department of Internal Medicine, Nagoya University School of Medicine, 65 Tsurumai, Showa-ku, Nagoya 466; [2]Department of Internal Medicine, Shinshiro Municipal Hospital, 32-1 Kitahata, Shinshiro, Aichi 441-13; [3]Department of Hematology/Oncology, National Cancer Center Hospital East, 6-5-1 Kashiwanoha, Kashiwa, Chiba 277; [4]Department of Internal Medicine, Japanese Red Cross Nagoya First Hospital, 3-35 Michishita, Nakamura-ku, Nagoya 453; [5]Clinical Preventive Services, Nagoya University School of Medicine, 65 Tsurumai, Showa-ku, Nagoya 466, Japan

**Summary** Carboplatin clearance depends on the glomerular filtration rate (GFR), and Calvert's formula is frequently used to achieve a target area under the time vs concentration curve (mg ml$^{-1}$ min). Creatinine clearance is a substitute for GFR when creatinine values are determined by the Jaffé method, which is being replaced by the enzymatic method. When the enzymatic method is used, the corresponding creatinine clearance theoretically exceeds GFR, and the use of creatinine clearance as GFR in Calvert's formula results, accordingly, in overdosing of carboplatin. In this study, we have established a model for adjusting the creatinine clearance to offset this bias based on a relationship between creatinine values measured by the Jaffé method and by the enzymatic method: adjusted creatinine clearance (ml min$^{-1}$) = creatinine clearance (ml min$^{-1}$) × [serum creatinine (mg dl$^{-1}$)]/[serum creatinine (mg dl$^{-1}$) + 0.2]. Subsequently, we validated this model using the data from 35 lung cancer patients. Estimated clearances of carboplatin with the original equation [creatinine clearance + 25] were systematically higher than observed clearances [mean prediction error (MPE) ± standard error (s.e.) = 26 ± 5%]. This positive bias was corrected by the adjustment (MPE ± s.e. = 5 ± 4%). When the enzymatic method is used, the adjusted creatinine clearance should be used in Calvert's formula.

**Keywords:** carboplatin; pharmacokinetics; chemotherapy; glomerular filtration rate; creatinine clearance

Carboplatin clearance depends on the glomerular filtration rate (GFR), and Calvert's formula is frequently used for determination of the dosing: dose (mg) = target AUC × [GFR (ml min$^{-1}$) + 25], where AUC denotes the area under the time vs concentration curve (mg ml$^{-1}$ min) (Harland et al, 1984; Calvert et al, 1989). In developing this formula, the GFR has been measured using the [$^{51}$Cr]EDTA clearance method. However, many oncologists have used creatinine clearance as a measure of GFR in the clinical use of this formula (Green and Smith, 1990; Sessa et al, 1991; Jodrell et al, 1992; Reyno et al, 1993; Langer et al, 1995), because obtaining the creatinine clearance is more convenient than measuring GFR directly. However, creatinine is both filtered by glomeruli and secreted by renal tubules, so the creatinine clearance theoretically exceeds GFR, thereby causing carboplatin overdosing (Doolan et al, 1962; Kassirer, 1971; Shemesh et al, 1985; Levey et al, 1991). We have reported more than 20% overdosing by replacing GFR with the creatinine clearance in this formula (Ando et al, 1997).

At present, two methods are available for measuring creatinine levels, the Jaffé method and the enzymatic method (Doolan et al, 1962; Fabiny and Ertingshausen, 1971; Kassirer, 1971; Larsen, 1972; Lustgarten and Wenk, 1972; Guder and Hoffmann, 1986; Crocker et al, 1988). The Jaffé method is based on a picrate reaction with creatinine under alkaline conditions and has been widely used for a long time with several modifications to the detailed procedure (Doolan et al, 1962; Fabiny and Ertingshausen, 1971; Larsen, 1972; Lustgarten and Wenk, 1972). This method is known to overestimate the serum level of creatinine by 5–15% because of a reaction with non-creatinine chromogens in serum but not in urine (Doolan et al, 1962; Kassirer, 1971). As a result of this bias, the true creatinine clearance is underestimated and can be accepted as a useful measure of GFR in clinical practice as this error coincidentally offsets the excess of creatinine clearance over GFR (Levey et al, 1991). On the other hand, the new enzymatic method is more specific and ensures better interlaboratory agreement than the traditional Jaffé method (Guder and Hoffmann, 1986; Crocker et al, 1988). Because the enzymatic method is not influenced by chromogens, the serum creatinine level is lower than when using the Jaffé method, and the corresponding creatinine clearance would be higher than GFR (Guder and Hoffmann, 1986; Crocker et al, 1988; Weber and van Zanten, 1991; Sokoll et al, 1994). Hence, the replacement of GFR with creatinine clearance in Calvert's formula causes carboplatin overdosing when the enzymatic method is used. This would explain the overestimation of carboplatin clearance in our previous study in which the enzymatic method was used.

We have attempted to evaluate the relationship between the creatinine levels determined using the Jaffé method and those determined using the enzymatic method. We have thus established a model to adjust the creatinine clearance by the enzymatic method to be used as GFR in Calvert's formula for the accurate prediction of the carboplatin clearance.

*Received 15 January 1997*
*Revised 24 March 1997*
*Accepted 4 April 1997*

*Correspondence to:* Y Ando, Division of Drug Metabolism, Faculty of Pharmaceutical Sciences, Hokkaido University, Kita-12, Nishi-6, Kita-ku, Sapporo 060, Japan

**Table 1** Patient characteristics and observed AUC

| | | |
|---|---|---|
| Regimen[a] | A/B/C | 14/11/10 |
| Gender | Female/male | 9/26 |
| Age (years) | Median | 61 (34–81) |
| Serum creatinine (mg $dl^{-1}$) | Mean ± s.d. | 0.8 ± 0.4 |
| | Median (range) | 0.7 (0.5–2.7) |
| Creatinine clearance (ml $min^{-1}$)[b] | Mean ± s.d. | 92 ± 29 |
| | Median (range) | 91 (12–154) |
| Weight (kg) | Median (range) | 50 (41–85) |
| Body surface area ($m^2$) | Median (range) | 1.49 (1.30–1.89) |
| Total dose of carboplatin (mg) | Median (range) | 514 (201–1162) |
| Observed AUC (mg $ml^{-1}$ min) | Median (range) | 5.75 (2.53–10.47) |

[a]Carboplatin was administered alone in regimen A and B, and with irinotecan in regimen C. [b]Creatinine values were all determined using the enzymatic method. AUC, area under the concentration time curve; s.d. standard deviation.

**Table 2** Chemotherapy regimens

| | |
|---|---|
| Regimen A | |
| Dose (mg) | Target AUC × [creatinine clearance (ml $min^{-1}$) + 25] |
| Infusion time | 60 min |
| Sampling | 0, 0.25, 0.5, 1, 2, 4, 8 h |
| Regimen B | |
| Dose (mg) | Target AUC × [0.76 × creatinine clearance (ml $min^{-1}$) + 15] |
| Infusion time | 60 min |
| Sampling | 0, 0.25, 0.5, 1, 1.5, 2, 4, 6, 8, 12, 24 h |
| Regimen C | |
| Dose (mg $m^{-2}$) | 300 |
| Infusion time | 90 min after irinotecan infusion |
| Sampling | 0, 0.25, 0.5, 1, 2.5, 4, 6.5, 22.5 h |

AUC, area under the concentration vs time curve (mg $ml^{-1}$ min).

### METHODS

### Relationship between the Jaffé method and the enzymatic method

We measured creatinine levels in serum and urine of the patients admitted to Shinshiro Municipal Hospital from November 1995 to March 1996. Diseases of these patients included various cancers, chronic obstructive pulmonary disease, ischaemic heart diseases, stable diabetes and chronic renal failure. Haemolysed samples or those from patients receiving any kind of antibiotics or vitamins were excluded (Lustgarten and Wenk, 1972; Guder and Hoffmann, 1986; Crocker et al, 1988; Weber and van Zanten, 1991). A urine sample from 24-h urine collection was obtained in the morning when the patient's serum sample was taken. The samples were stored at –20°C until analysis.

The serum and urine creatinine levels were determined using both the Jaffé method and the enzymatic method. The kinetic Jaffé method without deproteinization was performed with creatinine-HR (Wako Pure Chemical Industries, Osaka) using the auto-analyser Hitachi 747-400. The intra- and inter-day coefficient variation (CV) of the assay was under 3% and 6% respectively. The enzymatic method was carried out with Determiner-L CRE (Kyowa Medex, Tokyo) using Hitachi 717. The intra- and inter-day CV was less than 2% and 3% respectively. We developed equations using linear regression to convert creatinine values by the enzymatic method to those by the Jaffé method. Based on these equations, a model was developed to adjust creatinine clearance so as to be similar to GFR.

### Carboplatin pharmacokinetics

The pharmacokinetic data were available from 35 lung cancer patients (Table 1). They were treated with three different regimens including carboplatin (Table 2). Carboplatin was infused as monotherapy with the target AUC (mg $ml^{-1}$ min) of seven for untreated or five for previously treated patients in regimens A or B (Calvert et al, 1989). No antineoplastic treatment was allowed during the 4 weeks before entry for previously treated patients (3 months for cisplatin-containing regimen) (Daugaard et al, 1988). In regimen C, previously untreated patients received irinotecan at a dose ranging from 40 to 60 mg $m^{-2}$ followed by carboplatin. Creatinine clearance from 24-h urine collection was measured three times in regimens A and B or two times in regimen C, and the average value in each patient was used. Creatinine clearance was expressed as the absolute value in ml $min^{-1}$ without correction for the body surface area.

$$\text{Creatinine clearance (ml min}^{-1}) = \frac{[\text{urine volume (ml min}^{-1})] \times \text{urine creatinine (mg dl}^{-1})}{\text{serum creatinine (mg dl}^{-1})} \quad (1)$$

Blood samples were taken in a tube coated with a coagulation accelerant (Insepack, Sekisui Medical, Osaka) for pharmacokinetic analysis of carboplatin in regimens A and B, and the serum was immediately separated by centrifugation. Ultrafiltered serum was obtained using a Millipore Ultrafree-C3 filter unit (UFC3LGC00, Japan Millipore, Tokyo). In regimen C, plasma was obtained in heparinized tubes and was ultrafiltered with Amicon MPS micropartition system with YMT membranes (Grace Japan KK, Amicon, Tokyo). According to our data, the absolute difference in free platinum levels between the two procedures was 3.5 ± 0.8% (mean ± standard error, $n$ = 13). The ultrafiltered samples were stored at –20°C until analysis. All studies were approved by each institutional ethics committee, and written informed consent was obtained from all patients.

The ultrafiltered platinum level was measured by flameless atomic absorption spectrometry (LeRoy et al, 1977). The lower limit of detection was 25 ng $ml^{-1}$. The intra- and interassay CV was 2.6% and 4.1% respectively. The carboplatin level was calculated based on the molar ratio of platinum–carboplatin. Observed AUC was calculated by the trapezoidal method with extrapolation to infinity using PCNONLIN (version 4.0, Scientific Consulting, Apex, NC, USA). Estimated and observed clearances were calculated as follows:

$$\text{Estimated clearance (ml min}^{-1}) = \text{creatinine clearance (ml min}^{-1}) + 25$$

$$\text{Observed clearance (ml min}^{-1}) = \frac{\text{dose (mg)}}{\text{observed AUC (mg ml}^{-1}\text{ min)}}$$

All creatinine values had been determined by the enzymatic method. We recalculated the corresponding creatinine clearance in individual patients using the developed model. The accuracy of the clearance estimation was evaluated with the mean prediction error, its standard error (MPE ± s.e.) and the root mean squared error (RMSE) (Sheiner and Beal, 1981).

© Cancer Research Campaign 1997

**Table 3** Linear regression analysis of creatinine levels determined by the Jaffé method and the enzymatic method

| | Sample | | |
|---|---|---|---|
| | Serum | Serum | Urine |
| *n* | 85 | 73 | 32 |
| Range examined[a] (mg dl$^{-1}$) | 0.4–8.7 | 0.4–1.7 | 28.8–159.5 |
| Median | 0.9 | 0.8 | 57.35 |
| Slope[b] | 0.99 | 0.92 | 1.02 |
| 95% Confidence interval | 0.98–1.01 | 0.84–0.99 | 1.00–1.04 |
| Intercept[b] | 0.18 | 0.25 | - 0.04 |
| 95% Confidence interval | 0.15–0.22 | 0.18–0.32 | - 1.57–1.49 |
| *r* | 1.00 | 0.94 | 1.00 |

[a]The creatinine values were all determined by the enzymatic method.
[b]The values determined by the Jaffé method and those determined by the enzymatic method were dependent and independent variables respectively.

$$\text{MPE} = \frac{1}{n} \times \Sigma\ (\text{estimated} - \text{observed})\ (\text{ml min}^{-1})$$

$$= \frac{1}{n} \times \Sigma\ (\frac{\text{estimated} - \text{observed}}{\text{observed}} \times 100)\ (\%)$$

$$\text{RMSE} = [\ \frac{1}{n} \times \Sigma\ (\text{estimated} - \text{observed})^2]^{\frac{1}{2}}\ (\text{ml min}^{-1})$$

$$= [\ \frac{1}{n} \times \Sigma\ (\frac{\text{estimated} - \text{observed}}{\text{observed}} \times 100)^2]^{\frac{1}{2}}\ (\%)$$

### RESULTS

The linear regression analysis for serum creatinine levels between the Jaffé and enzymatic methods showed a slope of 0.99 and an intercept of 0.18, which was significantly different from zero (Table 3 and Figure 1). This implied that the values determined by the Jaffé method consistently exceeded those obtained by the enzymatic method for all creatinine levels investigated. To confirm that this relationship can be applied for the relevant levels to each dosing, we examined samples containing ≤ 1.7 mg dl$^{-1}$ as measured by the enzymatic method. The results were essentially the same (Table 3). Thus, when the enzymatic method was used to measure creatinine levels, serum creatinine in the denominator of the equation for creatinine clearance (eqn 1) should be replaced by serum creatinine + 0.2. On the other hand, good agreement was found in the urine creatinine levels by the two methods, and no modification was needed in the equation (Table 3 and Figure 1). Therefore, when creatinine levels were measured by the enzymatic method, GFR was estimated by adjusting the clearance as follows:

$$\text{Adjusted creatinine clearance (ml min}^{-1}) = \frac{[\text{urine volume (ml min}^{-1})] \times \text{urine creatinine (mg dl}^{-1})}{\text{serum creatinine (mg dl}^{-1}) + 0.2}$$

$$= \text{creatinine clearance (ml min}^{-1}) \times \frac{\text{serum creatinine (mg dl}^{-1})}{\text{serum creatinine (mg dl}^{-1}) + 0.2}$$

When the creatinine clearance was calculated with the values analysed by the enzymatic method and used in Calvert's formula, the estimated carboplatin clearance was positively biased and imprecise. After adjustment using the above model, this bias was significantly decreased ($P < 0.001$ by paired *t*-test; Table 4 and Figure 2). The precision expressed as RMSE was also improved (Table 4).

**Figure 1** Relationship between creatinine values analysed by the enzymatic method and by the Jaffé method in 85 serum (**A**) or 32 urine (**B**) samples. Lines denote the lines of identity

### DISCUSSION

The bias and precision of the carboplatin clearance estimation were improved by adjusting the creatinine clearance calculated with the values measured by the enzymatic method. This result confirmed our hypothesis that the use of the enzymatic method for serum creatinine determination overestimates carboplatin clearance when creatinine clearance is used as a substitute for GFR in Calvert's formula.

Recently, Chatelut et al (1995) proposed an alternative formula to calculate carboplatin clearance using the non-linear mixed-effect model (NONMEM), which included variables such as age, gender, weight and serum creatinine measured with an enzymatic

© Cancer Research Campaign 1997

Table 4 Observed and estimated clearances of carboplatin

| | Mean ± s.d. | MPE ± s.e. | RMSE |
|---|---|---|---|
| Observed (ml min⁻¹) | 97.5 ± 32.9 | | |
| Estimated | | | |
| Original (ml min⁻¹) | 117.3 ± 29.1 | 19.8 ± 4.2 | 31.3 |
| (%) | | (26 ± 5) | (37) |
| Adjusted (ml min⁻¹) | 96.5 ± 22.6 | − 1.0 ± 4.1 | 24.0 |
| (%) | | (5 ± 4) | (23) |

s.d., Standard deviation; MPE ± s.e., mean prediction error ± standard error; RMSE, root mean square error.

**Figure 2** Comparisons of the observed carboplatin clearances and those estimated before (**A**) or after (**B**) adjustment of creatinine clearance. Lines denote the lines of identity method (Chatelut et al, 1997). This model is beneficial when accurate measurement of GFR or creatinine clearance is difficult. However, in our study population, the Chatelut formula overestimated carboplatin clearance by $37 \pm 5$ ml min$^{-1}$ ($42 \pm 5\%$, MPE ± s.e.). There might be an ethnic difference in the utility of the Chatelut formula or a further as yet unestablished difference in the method of creatinine measurement.

This report recommends discontinuation of the Jaffé method for creatinine measurement, as it is readily influenced by non-creatinine chromogens and shows more interlaboratory variation (Doolan et al, 1962; Kassirer, 1971; Guder and Hoffmann, 1986). Instead, we suggest that creatinine levels should be measured by the enzymatic method while adjusting the creatinine clearance. This adjustment would lead to safer individualized dosing of carboplatin using Calvert's formula.

### ACKNOWLEDGEMENTS

This study was supported in part by a Grant-in-Aid for Cancer Research from the Ministry of Health and Welfare of Japan and Bristol-Myers Squibb, Nagoya, Japan. We wish to thank Masanori Moriya (Shinshiro Municipal Hospital), Kazuki Kadono (SRL, Aichi) and Nobuyuki Hosoya (Hachioji Labs SRL, Tokyo) for their assistance with sample management and the measurements.

### REFERENCES

Ando Y, Minami H, Ando M, Sugiura S, Sakai S, Saka H and Shimokata K (1997) Pharmacokinetic study of carboplatin given on a 5-day intravenous schedule. *Jpn J Cancer Res* **88**: 517–521

Calvert AH, Newell DR, Gumbrell LA, O'Reilly S, Burnell M, Boxall FE, Siddik ZH, Judson IR, Gore ME and Wiltshaw E (1989) Carboplatin dosage: prospective evaluation of a simple formula based on renal function. *J Clin Oncol* **7**: 1748–1756

Chatelut E, Canal P, Brunner V, Chevreau C, Pujol A, Boneu A, Roché H, Houin G and Bugat R (1995) Prediction of carboplatin clearance from standard morphological and biological patient characteristics. *J Natl Cancer Inst* **87**: 573–580

Chatelut E, Chevreau C and Canal P (1997) Prediction of carboplatin clearance from standard morphological and biological patient characteristics – response. *J Natl Cancer Inst* **89**: 261–262

Crocker H, Shephard MDS and White GH (1988) Evaluation of an enzymatic method for determining creatinine in plasma. *J Clin Pathol* **41**: 576–581

Daugaard G, Rossing N and Rørth M (1988) Effects of cisplatin on different measures of glomerular function in the human kidney with special emphasis on high-dose. *Cancer Chemother Pharmacol* **21**: 163–167

Doolan PD, Alpen EL and Theil GB (1962) A clinical appraisal of the plasma concentration and endogenous clearance of creatinine. *Am J Med* **32**: 65–79

Fabiny DL and Ertingshausen G (1971) Automated reaction-rate method for determination of serum creatinine with the CentrifiChem. *Clin Chem* **17**: 696–700

Green JA and Smith K (1990) Dose intensity of carboplatin in combination with cyclophosphamide or ifosfamide. *Cancer Chemother Pharmacol* **26**: s22–s25

Guder WG and Hoffmann GE (1986) Multicentre evaluation of an enzymatic method for creatinine determination using a sensitive colour reagent. *J Clin Chem Clin Biochem* **24**: 889–902

Harland SJ, Newell DR, Siddik ZH, Chadwick R, Calvert AH and Harrap KR (1984) Pharmacokinetics of cis-diammine-1,1-cyclobutane dicarboxylate platinum (II) in patients with normal and impaired renal function. *Cancer Res* **44**: 1693–1697

Jodrell DI, Egorin MJ, Canetta RM, Langenberg P, Goldbloom EP, Burroughs JN, Goodlow JL, Tan S and Wiltshaw E (1992) Relationships between carboplatin exposure and tumor response and toxicity in patients with ovarian cancer. *J Clin Oncol* **10**: 520–528

Kassirer JP (1971) Clinical evaluation of kidney function – glomerular function. *N Engl J Med* **285**: 385–389

Langer CJ, Leighton JC, Comis RL, O'Dwyer PJ, McAleer CA, Bonjo CA, Engstrom PF, Litwin S and Ozols RF (1995) Paclitaxel and carboplatin in combination in the treatment of advanced non-small-cell lung cancer: a phase II toxicity, response, and survival analysis. *J Clin Oncol* **13**: 1860–1870

Larsen K (1972) Creatinine assay by a reaction-kinetic principle. *Clin Chim Acta* **41**: 209–217

LeRoy AF, Wehling ML, Sponseller HL, Friauf WS, Solomon RE and Dedrick RL (1977) Analysis of platinum in biological materials by flameless atomic absorption spectrophotometry. *Biochem Med* **18**: 184–191

© Cancer Research Campaign 1997

Levey AS, Madaio MP and Perrone RD (1991) Laboratory assessment of renal disease: clearance, urinalysis, and renal biopsy. In *The Kidney*, 4th edn, Brenner BT and Rector FC Jr. (eds), pp. 919–968. WB Saunders: Philadelphia

Lustgarten JA and Wenk RE (1972) Simple, rapid, kinetic method for serum creatinine measurement. *Clin Chem* **18**: 1419–1422

Reyno LM, Egorin MJ, Canetta RM, Jodrell DI, Swenerton KD, Pater JL, Burroughs JN, Novak MJ and Sridhara R (1993) Impact of cyclophosphamide on relationships between carboplatin exposure and response or toxicity when used in the treatment of advanced ovarian cancer. *J Clin Oncol* **11**: 1156–1164

Sessa C, Goldhirsh A, Martinelli G, Alerci M, Imburgia L and Cavalli F (1991) Phase I study of the combination of monthly carboplatin and weekly cisplatin. *Ann Oncol* **2**: 123–129

Sheiner LB and Beal SL (1981) Some suggestions for measuring predictive performance. *J Pharmacokinet Biopharm* **9**: 503–512

Shemesh O, Golbetz H, Kriss JP and Myers BD (1985) Limitations of creatinine as a filtration marker in glomerulopathic patients. *Kidney Int* **28**: 830–838

Sokoll LJ, Russell RM, Sadowski JA and Morrow FD (1994) Establishment of creatinine clearance reference values for older women. *Clin Chem* **40**: 2276–2281

Weber JA and Van Zanten AP (1991) Interferences in current methods for measurements of creatinine. *Clin Chem* **37**: 695–700

© Cancer Research Campaign 1997